# Supplementary material for: A fault-intrusion-tolerant system and deadline-aware algorithm for scheduling scientific workflow in the cloud
Source: PeerJ Comput Sci. 2021 Nov 2;7:e747. doi: 10.7717/peerj-cs.747 (PMC8576554; doi:10.7717/peerj-cs.747)
Supplement: Supplemental Information 6 [file peerj-cs-07-747-s006.zip › Myproject1.1/docs/org/cloudbus/cloudsim/NetworkTopology.html]

NetworkTopology


JavaScript is disabled on your browser.


- Overview
- Package
- Class
- Use
- Tree
- Deprecated
- Index
- Help

- Prev Class
- Next Class

- Frames
- No Frames

- All Classes

- Summary:
- Nested |
- Field |
- Constr |
- Method

- Detail:
- Field |
- Constr |
- Method


org.cloudbus.cloudsim

## Class NetworkTopology

- java.lang.Object
- - org.cloudbus.cloudsim.NetworkTopology

- ---

    

  ```
  public class NetworkTopology
  extends java.lang.Object
  ```

  NetworkTopology is a class that implements network layer in CloudSim. It reads a BRITE file and
  generates a topological network from it. Information of this network is used to simulate latency
  in network traffic of CloudSim.

  The topology file may contain more nodes the the number of entities in the simulation. It allows
  for users to increase the scale of the simulation without changing the topology file.
  Nevertheless, each CloudSim entity must be mapped to one (and only one) BRITE node to allow
  proper work of the network simulation. Each BRITE node can be mapped to only one entity at a
  time.

  Since:
  :   CloudSim Toolkit 1.0

- - ### Field Summary

    Fields

    | Modifier and Type | Field and Description |
    | --- | --- |
    | `protected static double[][]` | `bwMatrix` |
    | `protected static DelayMatrix_Float` | `delayMatrix` |
    | `protected static TopologicalGraph` | `graph` |
    | `protected static java.util.Map<java.lang.Integer,java.lang.Integer>` | `map` |
    | `protected static int` | `nextIdx` |
  - ### Constructor Summary

    Constructors

    Constructor and Description

    `NetworkTopology()`
  - ### Method Summary

    Methods

    | Modifier and Type | Method and Description |
    | --- | --- |
    | `static void` | `addLink(int srcId, int destId, double bw, double lat)` Adds a new link in the network topology |
    | `static void` | `buildNetworkTopology(java.lang.String fileName)` Creates the network topology if file exists and if file can be succesfully parsed. |
    | `static double` | `getDelay(int srcID, int destID)` Calculates the delay between two nodes |
    | `static boolean` | `isNetworkEnabled()` This method returns true if network simulation is working. |
    | `static void` | `mapNode(int cloudSimEntityID, int briteID)` Maps a CloudSim entity to a node in the network topology |
    | `static void` | `unmapNode(int cloudSimEntityID)` Unmaps a previously mapped CloudSim entity to a node in the network topology |

    - ### Methods inherited from class java.lang.Object

      `clone, equals, finalize, getClass, hashCode, notify, notifyAll, toString, wait, wait, wait`

- - ### Field Detail


    - #### nextIdx

      ```
      protected static int nextIdx
      ```


    - #### delayMatrix

      ```
      protected static DelayMatrix_Float delayMatrix
      ```


    - #### bwMatrix

      ```
      protected static double[][] bwMatrix
      ```


    - #### graph

      ```
      protected static TopologicalGraph graph
      ```


    - #### map

      ```
      protected static java.util.Map<java.lang.Integer,java.lang.Integer> map
      ```
  - ### Constructor Detail


    - #### NetworkTopology

      ```
      public NetworkTopology()
      ```
  - ### Method Detail


    - #### buildNetworkTopology

      ```
      public static void buildNetworkTopology(java.lang.String fileName)
      ```

      Creates the network topology if file exists and if file can be succesfully parsed. File is
      written in the BRITE format and contains topologycal information on simulation entities.

      Parameters:
      :   `fileName` - name of the BRITE file


    - #### addLink

      ```
      public static void addLink(int srcId,
                 int destId,
                 double bw,
                 double lat)
      ```

      Adds a new link in the network topology

      Parameters:
      :   `srcId` - ID of the link's source
      :   `destId` - ID of the link's destination
      :   `bw` - Link's bandwidth
      :   `lat` - link's latency


    - #### mapNode

      ```
      public static void mapNode(int cloudSimEntityID,
                 int briteID)
      ```

      Maps a CloudSim entity to a node in the network topology

      Parameters:
      :   `cloudSimEntityID` - ID of the entity being mapped
      :   `briteID` - ID of the BRITE node that corresponds to the CloudSim entity


    - #### unmapNode

      ```
      public static void unmapNode(int cloudSimEntityID)
      ```

      Unmaps a previously mapped CloudSim entity to a node in the network topology

      Parameters:
      :   `cloudSimEntityID` - ID of the entity being unmapped


    - #### getDelay

      ```
      public static double getDelay(int srcID,
                    int destID)
      ```

      Calculates the delay between two nodes

      Parameters:
      :   `srcID` - ID of the source node
      :   `destID` - ID of the destination node

      Returns:
      :   communication delay between the two nodes


    - #### isNetworkEnabled

      ```
      public static boolean isNetworkEnabled()
      ```

      This method returns true if network simulation is working. If there were some problem during
      creation of network (e.g., during parsing of BRITE file) that does not allow a proper
      simulation of the network, this method returns false.

      Returns:
      :   $true if network simulation is ok. $false otherwise


- Overview
- Package
- Class
- Use
- Tree
- Deprecated
- Index
- Help

- Prev Class
- Next Class

- Frames
- No Frames

- All Classes

- Summary:
- Nested |
- Field |
- Constr |
- Method

- Detail:
- Field |
- Constr |
- Method
